# The influence of tactical formation on physical and technical performance across playing positions in the Chinese super league

**DOI:** 10.1038/s41598-024-53113-0

**Published:** 2024-01-30

**Authors:** Wei Zhang, Bo Gong, Rancheng Tao, Fei Zhou, Miguel Ángel Gómez Ruano, Changjing Zhou

**Affiliations:** 1 School of Athletic Performance, Shanghai University of Sport, Shanghai, 200438 China; 2 Facultad de Ciencias de la Actividad Física y del Deporte, Universidad Politécnica de Madrid, 28040 Madrid, Spain

**Keywords:** Physiology, Psychology

## Abstract

This study aimed to investigate the impact of tactical formations on the physical and technical performance of professional football players in the Chinese Super League (CSL). A sample of 800 games from the 2015–2021 CSL was analyzed, and players' physical (total distance covered, distance covered while ball in play, number of sprints, sprint distance, and high/middle/low-speed running) and technical (gain/loss of possession, ball retention percentage, challenges, challenge success percentage, passes, and pass success percentage) performance was assessed across six team formations: 3-5-2 (n = 137), 4-3-3 (n = 77), 4-2-3-1 (n = 391), 4-4-2 (n = 257), 3-4-3 (n = 41), and 4-1-4-1 (n = 107). Linear mixed models were used to assess variations in performance indicators across positions and formations. The results demonstrated that central defenders traveled significantly more total and low-speed running distances in the 3-5-2 formation than in the 4-2-3-1 formation (ES range: 0.33–0.34, p < 0.01). Fullbacks in the 3-5-2 formation demonstrated more high-speed running than did those in the 4-4-2 formation (ES = 0.27, p = 0.04). The central midfielders exhibited significantly more sprints and longer sprint distances in the 4-2-3-1 formation than in the 4-4-2 formation (ES range: 0.2–0.24, p < 0.01). Regarding technical performance, central defenders displayed significantly greater ball retention percentages, passes, and pass success rates in the 3-4-3 than in the 3-5-2 formations (ES range: 0.58–0.65, p < 0.01). Moreover, fullbacks and central midfielders executed markedly more passes with superior pass success rates in 4-back formations than in 3-5-2 formations (ES range: 0.2–0.53, p < 0.01). These findings can help coaches and academic staff understand the physical and technical requirements of various positions in various tactical formations, thus optimizing the training process.

## Introduction

Performance analysis has become an essential tool for academic researchers, sports organizations, athletes, and coaches. Collecting and interpreting performance data enables coaches to optimize their training programs, athletes to make better-informed tactical decisions, sports organizations to manage their teams more effectively, and researchers to gain a comprehensive understanding of sports performance^1^. In soccer, success is a result of effective tactics combined with the appropriate level of physical and technical performance^2^. However, analyzing physical and technical variables in isolation can limit our understanding and application of research findings in soccer^3^. This is because a player's performance in this sport is influenced not only by their physical and technical characteristics but also by mental and especially tactical factors^4^. The playing position and tactical formation are two of the most important tactical factors^5^. Thus, an integrated approach that examines physical, technical, and tactical indicators across different positions and formations is essential for comprehensively understanding the development of soccer matchplay^2^.

A growing body of research suggests that playing position can significantly affect physical and technical performance in soccer^6–9^. Specifically, central midfielders cover the greatest total distances, while wide midfielders and fullbacks achieve the greatest sprinting distances^10,11^. Wingbacks perform the highest number of accelerations^11^. These findings provide valuable insight into position-specific physical demands and could inform training programs for elite soccer players. Regarding technical performance, previous research has shown that, compared to other positions, midfielders make more passes and have higher passing success rates, while forwards take more shots but lose more duels and commit more turnovers, and defenders win a higher percentage of duels; additionally, fullbacks and wide midfielders make more crosses^8,12,13^. These variations in running and technical performance between positions are likely influenced by the various tactical roles that players take on during the game^13,14^. However, directly comparing performance indicators between positions fails to account for team tactics, which comprise an important contextual factor.

Over the years, coaches, players, and fans have discussed the best and most effective tactical team formation in soccer. Common tactical formations include the 4-4-2, 4-2-3-1, 4-1-4-1, 4-3-3, and 3-5-2 formations. However, few studies have specifically investigated the effects of various tactical formations on game performance^15^. Recently, Forcher et al.^16^ reported that formation has an impact on soccer players' physical and technical performance at both the team and position levels. They observed smaller differences at the team level for formations with a similar number of players per playing position (e.g., 4-5-1, 4-2-3-1). Additionally, their findings revealed that three-defender formations (e.g., 3-5-2) impose greater physical demands on players than four-defender formations (e.g., 4-4-2). These results are consistent with previous research conducted by Tierney et al.^17^, who compared five common playing formations (4-4-2, 4-2-3-1, 4-3-3, 3-4-3, and 3-5-2) and concluded that the 3-5-2 formation is more physically demanding than the other four formations. Additionally, concerning the differences in technical indicators between formations, Bradley et al.^18^ utilized a multicamera computer tracking system to analyze 20 English Premier League matches and discovered differences in technical indicators between formations. Specifically, players in the 4-4-2 and 4-3-3 formations completed more passes than did those in the 4-5-1 formation. Moreover, the percentage of successful passes was greater for the 4-4-2 formation than for the 4-3-3 and 4-5-1 formations.

However, these previous studies all have one limitation; i.e., they examined only the impact of tactical formation and playing position on match performance. The combination of formation and playing position appears to have more potential for providing a more comprehensive understanding of match performance^19,20^. To address this knowledge gap, Forcher et al.^5^ conducted a study on the 2018/19 season of the German Bundesliga. The study demonstrated that center backs and fullbacks covered the highest total and high-intensity distances in the 3-4-3 and 3-5-2 formations, respectively; wide midfielders displayed maximal values for these metrics in the 4-4-2 diamond but minimal values in the 3-4-3 formation; and central midfielders and forwards showed little variance across formations. While providing valuable insights, their findings were limited to one season in a single league, lacking longitudinal data across multiple seasons. As an analysis of football matches over time has shown, players' performance has changed tremendously over the years^21^. Examining data across multiple seasons enables a more precise assessment of the impacts of formation and position on match performance and determines the consistency of these impacts over an extended period^2,22^.

The Chinese Super League (CSL) has attracted increasing amounts of research attention, as it focuses on physical and technical metrics^23–25^. However, the influence of tactical factors remains unexplored. Therefore, this study aimed to investigate the specific impacts of different tactical formations (e.g., 4-4-2, 4-3-3, and 3-5-2) on physical indicators such as total distance covered and technical indicators such as the pass success rate among professional football players in different positions (e.g., defender, midfielder, forward) using longitudinal data from the 2015–2021 Chinese Super League seasons. The findings will not only enrich match analysis theories in football but also offer practical tactical insights for CSL coaches and administrators to improve Chinese professional football. Furthermore, it can assist in comprehending the physical and technical requirements of various positions for different tactical formations, ultimately optimizing the training process.

## Materials and methods

### Participants and design

The participants in this study were elite football players from teams competing in the CSL. The data for this study were obtained from 1364 matches in the past seven seasons (2015–2021) of the CSL; 564 matches with formation changes were excluded, and 800 matches without formation changes were ultimately included in the study. The teams’ starting formations in these matches were as follows: 3-5-2 (n = 137), 4-3-3 (n = 77), 4-2-3-1 (n = 391), 4-4-2 (n = 257), 3-4-3 (n = 41), and 4-1-4-1 (n = 107). The players’ physical and technical performance data were collected from teams that played in the 3-5-2, 4-4-2, 4-2-3-1, 4-1-4-1, 3-4-3, or 4-3-3 formations.

The analysis considered only the results of players who played for at least half of the match, excluding goalkeepers due to the specificity of the playing position. Given that changes in positional roles can impact a player's performance (e.g., physical and technical indicators), the analysis excluded players who switched their tactical roles^7,26^. Additionally, matches that included red cards were not analyzed. Players were classified as central defenders (CDs), fullbacks (FBs), central midfielders (CMs), (FWs), or wide midfielders (WMs) according to previous studies^5,11^. Among the 3-4-3 formations (CD: n = 116; FB: n = 87; CM: n = 80; and FW: n = 120), the 3-5-2 formations (CD: n = 419; FB: n = 282; CM: n = 426; and FW: n = 286), the 4-3-3 formations (CD: n = 157; FB: n = 175: CM: n = 237; and FW: n = 225), the 4-4-2 formations (CD: n = 520; FB: = 524; WM: n = 532; CM: n = 524; and FW: n = 526), the 4-1-4-1 formations (CD: n = 218; FB: n = 216; CM: n = 329; WM: n = 216; and FW: n = 113), and the 4-2-3-1 formations (CD: n = 789; FB: n = 795; CM: n = 1190; WM: n = 820; FW: n = 410). Due to the particularity of the positions of wide players in the 3-4-3 and 3-5-2 formations, we compared their running and technical indicators with those of wide midfielders and fullbacks in four-defender formations, such as 4-2-3-1 and 4-4-2. This approach involved investigating, from a macro perspective, the physical and technical requirements of wide players in different formations^11^. The study design and procedures were in accordance with the Declaration of Helsinki and approved by the ethics committee at Shanghai University of Sport. This study utilized publicly available match data for analysis and did not involve the collection of players' personal information. Therefore, informed consent was not obtained.

### Procedure

The analysis considered only teams that maintained a consistent formation throughout the entire match, as suggested previously^5,26,27^. In two stages, the consistency of the team formations was examined. In the first stage, the tactical format for each team and match was determined using official match reports from the CSL, which were provided by Amisco Pro^®^ (Amisco, Nice, France). The accuracy, validity, and reliability of the working process of Amisco Pro^®^ have been thoroughly discussed in prior studies^28,29^. In the second stage, we conducted a secondary verification of the average player positioning reported in official match reports against the match formations provided by a publicly accessible football statistics website known as "whoscored.com" (http://www.whoscored.com), whose original data from the OPTA Sportsdata company were tested for a respectable level of interoperator reliability (Kappa values > 0.90)^30^.

The physical and technical data of players in different positions in various formations were collected by a semiautomatic computerized video tracking system, Amisco Pro^®^ (Amisco, Nice, France). The physical variables included the following: total distance covered (km), distance covered while ball in play (km), number of sprints, sprint distance (25.1–> km/h), high-speed running (19.7–> 25.1 km/h), middle-speed running (14.3–> 19.7 km/h), and low-speed running (7.1–> 14.3 km/h). Additionally, the technical variables included the following: gain of possession, loss of possession, ball retention percentage, challenges, challenge success percentage, passes, and pass success percentage. The operational definitions of the technical and physical performance-related parameters are presented in Table 1.

**Table 1:** Technical and physical performance-related parameters (dependent variables).

Physical performance-related parameters: operational definition
Total distance covered (km): Distance covered in a match
Low speed running: Distance covered at a speed of 7.1- > 14.3 km/h in a match
Middle speed running: Distance covered at a speed of 14.3- > 19.7 km/h in a match
High speed running: Distance covered at a speed of 19.7- > 25.1 km/h in a match
Sprint: Distance covered at a speed over 25.1 km/h in a match
Number of sprints: Number of sprints covered at the speed over 25.1 km/h in a match
Distance covered ball in play (km): Distance covered when the ball was in play
Technical performance-related parameters: operational definition
Pass: an intentional played ball from one player to another
Pass success percentage: successful passes as a proportion of total passes
Challenge: actions when two players are competing for ball possession, which is not in the control of any player, i.e., both players have approximately a 50% chance of gaining control of the ball; includes ground and air challenges
Challenge success percentage: successful challenges as a proportion of the total challenges
Ball retention percentage: ball retention percentage refers to the percentage of time a player to keep possession of the ball during a match
Gain of possession: the action of gaining possession from an opposition player who is in possession of the ball
Loss of possession: the moment when a player who had control of the ball loses it to an opponent, either through a mistake or due to the opponent's skillful intervention

### Statistical analysis

The Kolmogorov‒Smirnov test was used to ensure that the data were normally distributed, and the data are displayed as the means ± standard deviations. Levene's test verified the homoscedasticity of all the variables. Linear mixed models for repeated measures were used to examine the differences in the physical and technical performance of players at different positions in different formations. The formation was used as a fixed effect, the players were used as a random effect to account for repeated measures, and the physical and technical indicators were used as dependent variables. Finally, Bonferroni post hoc multiple comparisons were performed to check for significant differences. To interpret the magnitude of differences, Cohen's d was used to calculate effect sizes (ESs) and was interpreted as follows: < 0.2, trivial; 0.2–0.6, small; 0.6–1.2, medium; 1.2–2.0, large; and > 2.0, very large^31^. The level of statistical significance was set at p < 0.05. Statistical analyses were performed using R statistical software (version 3.4.3; R Foundation for Statistical Computing, Vienna, Austria)^3^.

## Results

Tables 2 and 3 show descriptive statistics for the means and standard deviations of physical and technical indicators for each player position in different formations.

**Table 2:** Means ± SDs of physical variables for the different playing styles by positional role.

Variable	Position	3-4-3	3-5-2	4-3-3	4-4-2	4-1-4-1	4-2-3-1	p value	
Total distance covered (km)	CD	9.43 ± 0.66	9.49 ± 0.83^#^	9.39 ± 0.64	9.20 ± 0.78	9.39 ± 0.71	9.24 ± 0.70		P = 0.005
	FB	10.43 ± 0.99^$#^	10.52 ± 0.83^cd$#^	10.22 ± 0.78	10.18 ± 0.83	10.21 ± 0.65	10.20 ± 0.73		P < 0.001
	CM	10.88 ± 0.77	11.21 ± 1.15^c#^	10.72 ± 0.84	10.79 ± 0.80	10.84 ± 0.77	10.69 ± 0.85		P = 0.001
	WM	10.43 ± 0.99	10.52 ± 0.83		10.92 ± 0.95^ab#^	10.62 ± 0.90	10.59 ± 0.90		P < 0.001
	FW	10.27 ± 0.89	10.28 ± 1.20^#^	10.23 ± 0.94	10.23 ± 0.96^#^	9.92 ± 0.89	9.89 ± 1.03		P = 0.03
Distance covered ball in play (km)	CD	6.66 ± 0.75^#^	6.48 ± 0.78^#^	6.40 ± 0.62	6.31 ± 0.76	6.49 ± 0.70	6.30 ± 0.74		P < 0.001
	FB	7.37 ± 0.92^d$#^	7.19 ± 0.87^#^	7.05 ± 0.81	7.04 ± 0.82	7.09 ± 0.77	7.01 ± 0.79		P < 0.001
	CM	7.95 ± 0.92	7.97 ± 1.03^#^	7.62 ± 0.94	7.68 ± 0.87	7.78 ± 0.96	7.54 ± 0.92		P < 0.001
	WM	7.37 ± 0.92	7.19 ± 0.87		7.61 ± 0.96^b#^	7.40 ± 0.96	7.30 ± 0.94		P < 0.001
	FW	7.34 ± 1.02^#^	7.11 ± 1.08^#^	7.10 ± 0.92	7.10 ± 0.89^#^	6.92 ± 0.92	6.77 ± 0.98		P < 0.001
Number of sprints	CD	5.02 ± 3.13	4.23 ± 2.76	4.06 ± 2.36	4.29 ± 2.68	4.21 ± 2.52	4.24 ± 2.56		P = 0.09
	FB	8.86 ± 4.50	7.81 ± 4.18	8.16 ± 3.57	7.86 ± 3.82	7.70 ± 3.94	8.16 ± 3.86		P = 0.34
	CM	5.13 ± 3.42	6.03 ± 4.42	5.21 ± 3.03	4.70 ± 2.95	5.16 ± 3.43	5.54 ± 3.67^d^		P = 0.009
	WM	8.86 ± 4.50	7.81 ± 4.18		9.67 ± 4.40^b^	10.11 ± 4.53^b^	9.93 ± 4.77^b^		P < 0.001
	FW	8.55 ± 4.44	9.44 ± 4.80	9.38 ± 4.43	8.48 ± 4.26	8.41 ± 4.84	7.95 ± 4.34		P = 0.15
Sprint	CD	101.47 ± 67.80	83.76 ± 61.46	79.82 ± 50.25	84.62 ± 56.14	84.07 ± 52.88	83.95 ± 56.67		P = 0.08
	FB	182.75 ± 103.76	157.94 ± 92.86	169.33 ± 81.27	161.68 ± 87.52	159.86 ± 87.65	168.21 ± 90.37		P = 0.37
	CM	96.66 ± 66.32	118.32 ± 92.49	101.25 ± 64.14	93.02 ± 64.76	100.02 ± 72.33	108.24 ± 79.01^d^		P = 0.01
	WM	182.75 ± 103.76	157.94 ± 92.86		200.17 ± 101.93^b^	207.09 ± 100.05^b^	207.61 ± 112.28^b^		P < 0.001
	FW	172.51 ± 93.76	197.69 ± 117.11	195.67 ± 103.73	177.30 ± 100.85	175.93 ± 114.70	162.33 ± 96.92		P = 0.39
High speed running	CD	361.85 ± 140.78	334.72 ± 27.26	340.76 ± 103.89	337.24 ± 121.90	330.08 ± 112.27	328.58 ± 108.68		P = 0.31
	FB	624.23 ± 173.58	610.26 ± 177.91^d^	583.05 ± 158.98	565.48 ± 159.79	572.51 ± 167.70	576.86 ± 158.90		P = 0.02
	CM	517.96 ± 173.58	610.20 ± 243.13	540.61 ± 185.61	518.87 ± 173.37	550.38 ± 187.23	542.95 ± 193.79		P = 0.18
	WM	624.23 ± 173.58	610.26 ± 177.91		729.75 ± 218.76^ab^	685.26 ± 201.54^b^	671.39 ± 200.28^ab^		P < 0.001
	FW	602.35 ± 188.95	595.26 ± 189.00	624.96 ± 182.65	600.10 ± 182.19	568.32 ± 179.97	538.41 ± 183.13		P = 0.18
Middle speed running	CD	927.47 ± 264.19	941.02 ± 267.94	944.88 ± 251.67	914.12 ± 228.18	968.73 ± 263.82	910.96 ± 227.72		P = 0.68
	FB	1389.71 ± 307.31^d$#^	1397.61 ± 334.01^cd$#^	1286.39 ± 285.17	1260.08 ± 276.54	1284.97 ± 246.50	1281.39 ± 265.68		P < 0.001
	CM	1637.96 ± 371.82	1744.67 ± 485.41	1555.35 ± 363.75	1608.86 ± 367.35	1631.83 ± 377.63	1554.30 ± 374.04		P = 0.50
	WM	1389.71 ± 307.31	1397.61 ± 334.01		1564.06 ± 378.97^ab^	1397.61 ± 365.51	1435.38 ± 346.58^a^		P < 0.001
	FW	1304.40 ± 361.85	1276.27 ± 448.44	1308.19 ± 349.41	1289.83 ± 364.18^#^	1181.51 ± 276.45	1194.84 ± 327.01		P = 0.009
Low speed running	CD	3561.60 ± 564.45	3750.50 ± 575.64^#^	3639.55 ± 533.69	3492.99 ± 611.21	3666.48 ± 600.28	3557.83 ± 599.30		P = 0.005
	FB	3874.83 ± 674.39	4100.18 ± 570.86^d$#^	3899.95 ± 597.74	3886.19 ± 631.05	3955.38 ± 548.93	3919.79 ± 566.59		P < 0.001
	CM	4591.48 ± 666.24	4652.80 ± 744.22	4361.20 ± 680.13	4457.35 ± 654.03^#^	4497.44 ± 639.88	4376.13 ± 696.45		P = 0.007
	WM	3874.83 ± 674.39	4100.18 ± 570.86		4161.91 ± 739.02	3999.56 ± 704.68	4018.72 ± 685.97		P = 0.09
	FW	3807.94 ± 714.93	3751.77 ± 941.54	3703.49 ± 726.02	3706.38 ± 771.74^#^	3599.95 ± 703.34	3590.67 ± 725.87		P = 0.018

*CD* central defender, *FB* fullback, *CM* central midfielder, *WM* wide midfielder, *FW* forward.

^a^Greater than their 3-4-3 counterparts, ^b^greater than their 3-5-2 counterparts, ^c^greater than their 4-3-3 counterparts, ^d^greater than their 4-4-2 counterparts, ^$^greater than their 4-1-4-1 counterparts, and ^#^greater than their 4-2-3-1 counterparts.

**Table 3:** Means ± SDs of technical variables for the different playing styles by positional role.

Variable	Position	3-4-3	3-5-2	4-3-3	4-4-2	4-1-4-1	4-2-3-1	p value
Gain of possession	CD	12.08 ± 4.10	12.87 ± 4.61	13.66 ± 4.57^a^	13.23 ± 4.43	13.19 ± 4.52	13.20 ± 4.37	P = 0.10
	FB	9.91 ± 3.74	10.19 ± 3.63	11.35 ± 4.24	11.58 ± 4.23^ab^	11.21 ± 3.57	11.49 ± 4.02^ab^	P < 0.001
	CM	11.14 ± 4.01	10.81 ± 4.63	10.88 ± 4.81	12.27 ± 4.81^$#^	10.29 ± 4.95	10.75 ± 5.16	P < 0.001
	WM	9.91 ± 3.74^d$#^	10.19 ± 3.63^d$#^		7.77 ± 3.69^$#^	6.58 ± 3.48	6.90 ± 3.38	P < 0.001
	FW	5.30 ± 2.99^$#^	4.68 ± 2.63	5.88 ± 3.44^bd$#^	4.83 ± 2.80	3.63 ± 2.37	4.09 ± 2.70	P < 0.001
Loss of possession	CD	9.04 ± 3.48	10.00 ± 4.03	9.94 ± 4.23	9.46 ± 3.90	9.97 ± 4.47	9.45 ± 3.90	P = 0.31
	FB	14.63 ± 5.34	13.90 ± 4.85	13.36 ± 5.06	13.70 ± 5.00	13.70 ± 4.75	13.66 ± 4.64	P = 0.58
	CM	12.92 ± 4.97	13.65 ± 5.38	12.82 ± 5.57	12.30 ± 4.96	12.34 ± 4.96	13.49 ± 5.51	P = 0.06
	WM	14.63 ± 5.34	13.90 ± 4.85		13.77 ± 5.29	13.85 ± 5.40	14.25 ± 4.86	P = 0.37
	FW	14.32 ± 5.06	14.73 ± 5.10	15.08 ± 5.86	15.00 ± 5.15	13.96 ± 4.44	14.54 ± 4.96	P = 0.42
Ball retention percentage	CD	80.17 ± 9.78^b^	73.43 ± 11.90	75.87 ± 11.93	75.79 ± 11.50	76.40 ± 11.30	76.05 ± 10.65	P = 0.04
	FB	73.91 ± 10.33	70.55 ± 10.05	75.25 ± 8.79^b^	74.00 ± 9.08^b^	74.36 ± 8.41^b^	74.87 ± 8.53^b^	P < 0.001
	CM	79.26 ± 8.31^b^	73.44 ± 9.80	77.74 ± 9.11^b^	77.46 ± 8.84^b^	77.58 ± 8.51^b^	76.72 ± 9 37^b^	P < 0.001
	WM	73.91 ± 10.33^d$#^	70.55 ± 10.05		69.15 ± 10.68	67.81 ± 9.89	68.68 ± 10.05	P = 0.006
	FW	66.28 ± 11.52	63.06 ± 11.08	65.00 ± 10.96	63.61 ± 10.55	62.51 ± 10.30	62.16 ± 10.25	P = 0.09
Challenge	CD	5.98 ± 3.36	6.10 ± 3.45	6.07 ± 3.35	6.22 ± 3.52	5.65 ± 3.49	6.12 ± 3.37	P = 0.34
	FB	4.17 ± 2.70	4.53 ± 2.69	4.94 ± 2.86	4.67 ± 2.63	4.72 ± 2.69	5.04 ± 2.83	P = 0.04
	CM	5.17 ± 3.35	5.79 ± 3.50	5.29 ± 3.39	5.51 ± 3.25	5.26 ± 2.92	6.20 ± 3.45^$^	P < 0.001
	WM	4.17 ± 2.70	4.53 ± 2.69		5.65 ± 3.28^ab^	5.71 ± 3.49^ab^	6.20 ± 3.49^ab^	P < 0.001
	FW	7.73 ± 5.11	10.14 ± 5.49	7.87 ± 5.00	9.47 ± 5.58	9.37 ± 4.69	11.51 ± 5.29^acd^	P < 0.001
Challenge success percentage	CD	55.12 ± 24.03	58.79 ± 25.25	59.92 ± 26.79	61.31 ± 24.40	60.83 ± 26.13	61.10 ± 25.15	P = 0.19
	FB	49.09 ± 32.13	50.06 ± 29.04	54.55 ± 29.09	52.37 ± 28.17	52.15 ± 28.46	54.35 ± 28.46	P = 0.23
	CM	53.48 ± 26.42	47.62 ± 27.22	48.98 ± 28.54	52.01 ± 26.69	49.97 ± 28.85	48.47 ± 26.19	P = 0.06
	WM	49.09 ± 32.13	50.06 ± 29.04^d$#^		41.66 ± 27.55	40.30 ± 27.83	41.49 ± 26.25	P < 0.001
	FW	37.85 ± 24.40	42.48 ± 20.79	46.23 ± 26.52^a^	44.26 ± 22.51	39.49 ± 21.00	42.35 ± 19.08	P = 0.021
Pass	CD	49.24 ± 16.94^b^	39.80 ± 15.99	44.19 ± 16.35	41.11 ± 14.77	45.17 ± 17.77	41.00 ± 14.49	P = 0.002
	FB	54.37 ± 16.80^b^	45.35 ± 14.40	53.10 ± 17.33^b^	51.37 ± 15.34^b^	52.30 ± 14.99^b^	53.22 ± 15.17^b^	P < 0.001
	CM	60.97 ± 20.37^b^	49.19 ± 20.18	55.61 ± 20.26^b^	53.08 ± 18.27^b^	53.31 ± 19.27^b^	55.24 ± 19.01^b^	P = 0.001
	WM	54.37 ± 16.80^d$#^	45.35 ± 14.40^d$#^		39.73 ± 15.27	36.03 ± 12.95	39.34 ± 13.64	P < 0.001
	FW	35.80 ± 14.82	31.60 ± 12.79	35.16 ± 13.61^$#^	34.2 ± 13.40	29.21 ± 10.14	30.50 ± 11.78	P = 0.002
Pass success percentage	CD	80.17 ± 10.06^b^	72.25 ± 12.77	75.61 ± 12.85	75.20 ± 12 33^b^	75.85 ± 11.74	75.26 ± 11.31	P < 0.001
	FB	72.88 ± 10.84	68.44 ± 10.52	73.63 ± 9.38^b^	72.25 ± 9.62^b^	72.54 ± 9.05^b^	72.70 ± 8.87^b^	P < 0.001
	CM	80.27 ± 8.07^b^	74.19 ± 10.49	78.67 ± 9.26^b^	78.13 ± 9.28^b^	78.89 ± 9.25^b^	77.60 ± 9.83^b^	P < 0.001
	WM	72.88 ± 10.84	68.44 ± 10.52		70.46 ± 11.44	69.83 ± 11.91	70.29 ± 11.00	P = 0.19
	FW	71.06 ± 11.79	67.19 ± 12.45	68.95 ± 11.81	67.65 ± 11.51	68.98 ± 12.03	67.24 ± 11.65	P = 0.32

*CD* central defender, *FB* fullback, *CM* central midfielder, *WM* wide midfielder, *FW* forward.

^a^Greater than their 3-4-3 counterparts, ^b^greater than their 3-5-2 counterparts, ^c^greater than their 4-3-3 counterparts, ^d^greater than their 4-4-2 counterparts, ^$^greater than their 4-1-4-1 counterparts, and ^#^greater than their 4-2-3-1 counterparts.

**Figure 1:**
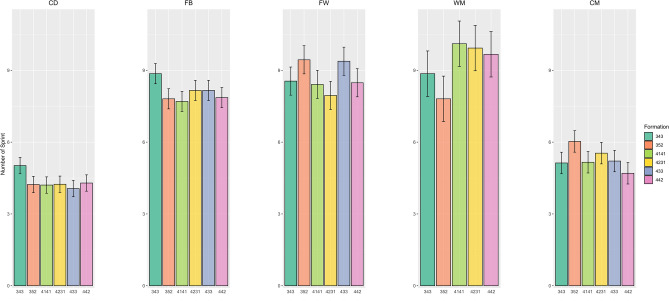
The number of sprints covered by players at different positions across different formations.

**Figure 2:**
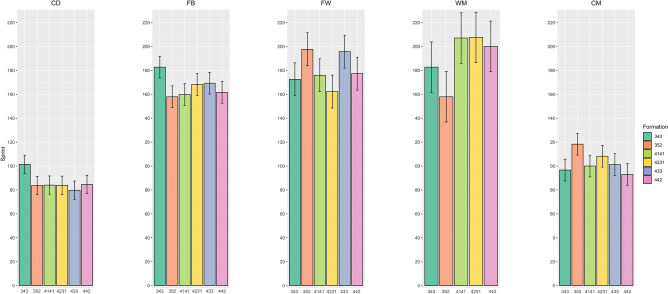
The sprint distance covered by players at different positions across different formations.

**Figure 3:**
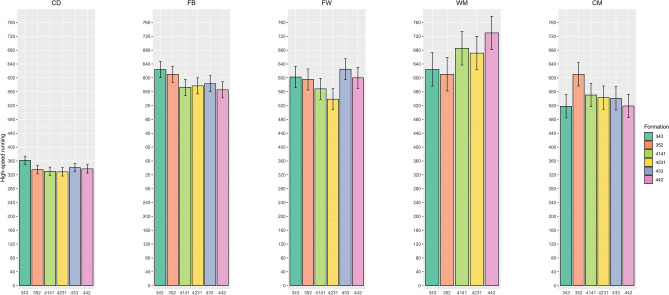
The high-speed running covered by players at different positions across different formations.

The means ± SDs of the physical variables for the different playing styles by positional role are presented in Table 2. Specifically, the total and low-speed running distances covered by central defenders in the 3-5-2 formation were greater than those in the 4-2-3-1 formation (ES range = 0.33–0.34, p < 0.01). Additionally, fullbacks in the 3-5-2 formation covered more total and moderate-speed running distances than did their counterparts in the 4-3-3 (ES range = 0.35–0.37, p < 0.01), 4-4-2 (ES range = 0.4–0.46, p < 0.01), 4-1-4-1 (ES range = 0.38–0.41, p < 0.01), and 4-2-3-1 (ES range = 0.41–0.43, p < 0.01) formations. Central midfielders in the 3-5-2 formation also travelled more total distance and were farther from the ball in play than were those in the 4-2-3-1 formation (ES range = 0.45–0.55, p < 0.01). Furthermore, there were significant differences found in the sprint distance and number of sprints between formations, with central midfielders in the 4-2-3-1 formation having more sprints and longer sprint distances than those in the 4-4-2 formation (ES range = 0.2–0.24, p < 0.01), as shown in Figs. 1 and 2. Wide midfielders also showed more sprints and longer sprint distances in the 4-4-2, 4-1-4-1, and 4-2-3-1 formations than in the 3-5-2 formation (ES range = 0.43–0.53, p < 0.01), as shown in Figs. 1 and 2. Moreover, Fig. 3 shows that fullbacks in the 3-5-2 formation demonstrated more high-speed running than did those in the 4-4-2 formation (ES = 0.27, p = 0.04). Finally, while forwards exhibited less pronounced differences in high-intensity activity (e.g., number of sprints, sprint distance, and high-speed running), they covered more total distance in the 3-5-2 and 4-4-2 formations than in the 4-2-3-1 formation (ES range = 0.34–0.35, p = 0.02) (Fig. 1, Fig. 2, Fig. 3).

The means ± SDs of the technical variables for the different playing styles by positional role are shown in Table 2. Regarding technical parameters, central defenders in the 3-4-3 formation outperformed their counterparts in the 3-5-2 formation (ES range = 0.58–0.65, p < 0.01) in terms of ball retention percentage, passes, and pass success rate. In these formations, 4-3-3, 4-4-2, 4-1-4-1, and 4-2-3-1, fullbacks and central midfielders made more passes and had a higher pass success rate than did those in the 3-5-2 formation (ES range = 0.2–0.53, p < 0.01). Wide midfielders in 4-4-2, 4-1-4-1, and 4-2-3-1 formations presented more challenges than did those in 3-4-3 (ES range = 0.46–0.59, p < 0.01) and 3-5-2 (ES range = 0.36–0.51, p < 0.01) formations; however, the challenge success rate associated with wide midfielders in the 3-5-2 formation was greater than that associated with the 4-4-2 (ES = 0.3, p < 0.01), 4-1-1 (ES = 0.34, p < 0.01), and 4-2-3-1 (ES = 0.32, p < 0.01) formations. The number of passes made by forwards in the 4-3-3 formation was significantly greater than that in the 4-1-4-1 (ES = 0.47, p = 0.03) and 4-2-3-1 (ES = 0.37, p = 0.01) formations; however, there was no significant difference found in in the pass success rate between the formations.

## Discussion

This study aimed to investigate the impact of different tactical formations on the physical and technical performance of players in various positions in the CSL. The findings demonstrate that significant variations exist in the physical and technical indicators of players across different positions, which could be attributed primarily to their tactical roles within diverse formations.

According to the current research, central defenders in the 3-5-2 formation cover a greater distance than those in the 4-2-3-1 formation. This finding aligns with a previous study on the UEFA Champions League, which also revealed that 3-5-2 formation leads to greater physical demands for central defenders^26^. This could indicate that, regardless of a league's level of competition, the 3-5-2 formation inherently requires central defenders to cover more distance. Similar findings have been reported for the German Bundesliga^5^, where 3-5-2 formations’ central defenders also demonstrate greater running output than that of those playing in 4-defender formations. Furthermore, from a football tactical perspective, central defenders' tactical roles and responsibilities differ between team formations consisting of three and four defensive players^26,32,33^, which could explain differences in running distance. Specifically, central defenders are highly involved in attacking actions in three-defender formations^34,35^. As a result, they must utilize the full width of the field when attacking by positioning themselves closer to the opponent's half. However, upon losing possession, central defenders must swiftly transition from offense to defense along with fullbacks to establish a robust defense and prevent conceding goals. This defensive and transitional style may reveal the need for central defenders to cover more distance in the field.

Regarding technical performance, the central defenders in the 3-4-3 formation exhibit superior performance metrics, such as ball retention percentage, passes, and pass success rate, compared to those in the 3-5-2 formation. This finding contradicts the findings from previous research on the Bundesliga, which have indicated that central defenders perform better technically in 4-3-3 and 4-2-3-1 formations than in other formations^5^. This disparity may be due to the relatively smaller database used in the previous study (one season)^36^ and to tactical differences between the leagues and teams analyzed^37^. Moreover, the divergent midfield configurations provide further explanation. Specifically, with three central midfielders organizing attacks, the 3-5-2 formation alleviates the attacking responsibilities on central defenders. By comparison, the 3-4-3 formation, with only two central midfielders, places greater demands on central defenders to initiate attacks by achieving high passing accuracy and ball retention. Therefore, compared to the 3-5-2 formation, the 3-4-3 formation demands a greater level of passing and ball retention from the central defenders.

Fullbacks (also called wingbacks in three-back formations), similar to central defenders, have different tactical roles when playing with three or four defensive players^26,32^. Specifically, compared to fullbacks in other formations such as the 4-4-2, 4-3-3, 4-1-4-1, and 4-2-3-1 formations, those in the 3-5-2 and 3-4-3 formations cover a greater total distance and a greater distance from the ball during play. This finding is consistent with previous findings regarding the Bundesliga^5^ and the Croatian League^35^. Additionally, it was observed that the fullbacks in the 3-5-2 formation exhibit more high-speed running than those in the 4-4-2 formation, while the fullbacks in the 3-4-3 formation exhibit more moderate-speed running than their counterparts in other formations. Therefore, based on the findings of our study and those of other studies^5,26,35,38^, fullbacks exhibit a greater level of running performance in formations with three central defenders than in formations with four defenders (e.g., 4-1-4-1 and 4-2-3-1). A plausible explanation for these results might be the absence of wide midfielders in the 3-5-2 and 3-4-3 formations, which require greater involvement of the fullbacks (wingbacks) both in the attacking and defending phases^39^. In more detail, when attacking, the fullbacks must support the team's overall attack by providing an attack width, often pushing into the opponent's defensive third. However, they must also quickly retreat into the defensive third when their team loses possession to form a back five with the three central defenders. As a result, fullbacks may perform offensive and defensive tasks with more running output.

In terms of technical indicators, it was discovered that fullbacks in formations such as 4-3-3, 4-4-2, 4-1-4-1, and 4-2-3-1 have higher ball retention percentages, more passes, and a higher rate of successful passes than those in the 3-5-2 formation. This difference can be attributed to the tactical roles of players in different formations^5,26^. Specifically, in three-defender formations such as 3-5-2, fullbacks tend to have reduced ball control duties, as central defenders assume greater possession responsibilities and are highly involved in attacking actions^34,35^. As a result, fullbacks (wingbacks) typically need to create opportunities for team attack through active running (e.g., more total distance, more high-speed running, and more moderate-speed running than fullbacks in four-back formations). However, in four-back formations, fullbacks play a critical role in the team's build-up play, requiring them to possess strong passing and individual ball control abilities. Therefore, it can be concluded that the 3-5-2 and 3-4-3 formations impose greater physical demands on fullbacks, while the 4-4-2, 4-3-3, 4-1-4-1, and 4-2-3-1 formations necessitate fullbacks with greater technical skill.

Team formation has a significant impact on the physical performance of central midfielders^26,40^. Specifically, the central midfielders in the 4-2-3-1 formation engage in more sprints and sprint greater distances than their counterparts in the 4-4-2 formation. Arjol-Serrano et al.^40^ reported that the 4-4-2 formation places less physical demand on central midfielders than does the 4-2-3-1 formation. In addition, studies on the German Bundesliga and UEFA Champions League have shown that the 4-4-2 formation places lower physical demands on central midfielders than other formations^5,26^. The specific organization and positioning of midfield players, such as central midfielders and wide midfielders, in different team formations may provide an explanation for this phenomenon^41^. In detail, compared to those of the 4-2-3-1 formation, the best feature of the 4-4-2 formation is its balance of offense and defense—i.e., midfield players have clear responsibilities, a clear division of labor and are closely linked to each other—resulting in greater compactness in the middle of the pitch^42^. Therefore, the reduced spatial coverage likely explains the fewer sprints and shorter sprint distances observed in central midfielders playing in the 4-4-2 formation.

Looking at the technical performance, central midfielders in the 3-5-2 formation have a lower ball retention percentage, a lower pass success rate, and fewer passes than their counterparts in the 4-4-2, 4-1-4-1, 4-3-3, and 4-2-3-1 formations. It is widely acknowledged that central midfielders play a crucial role in coordinating offensive actions^34,35^. However, in 3-5-2 formations, the responsibility of the central midfielders to organize the attack may be weakened as the central defenders take on more of the organizing role. This may explain why central midfielders in the 3-5-2 formation made fewer passes, had lower pass success rates, and had a lower ball retention percentage than did those in the four-back formations. In terms of challenge, the central midfielders in the 4-2-3-1 formation outperform their counterparts in the 4-1-4-1 formation. An explanatory approach could be that in the 4-2-3-1 formation, the midfield typically consists of two defensive midfielders and one attacking midfielders^26^, whereas in the 4-1-4-1 formation, the midfield consists of one defensive midfielder and two attacking midfielders. Due to the presence of two defensive midfielders in the 4-2-3-1 formation, the defense in the midfield will be more intense, and the central midfielders will exert more pressure on the opponent; therefore, the central midfielders faced more challenges than those in the 4-1-4-1 formation.

Regarding wide midfielders, we found that in the 4-4-2 formation, the total distance covered by wide midfielders is greater than that covered by their counterparts in other formations. This result is consistent with previous findings from studies on the German Bundesliga^5^ and the UEFA Champions League^26^. One probable explanation for this observation is that the 4-4-2 formation has a more balanced number of players in each position^43^. As a result, wide midfielders are crucial members of the team, both offensively and defensively. For example, during attacks, wide midfielders must make full use of the space on the flank to create scoring opportunities for the forwards (e.g., dribbling with crosses of the ball), whereas during defense, wide midfielders must return to the midfield to participate in the overall defense of the team. Unlike the 4-2-3-1 and 3-5-2 formations, the 4-4-2 formation has only two central midfielders; thus, the wide midfielders need to take on more defensive duties and cover more distance in the midfield area than the wide midfielders in other formations. In contemporary soccer, high-speed running or sprints are essential for successful performance^44–46^. In our research, we found that wide midfielders in the 4-4-2, 4-1-4-1, and 4-2-3-1 formations exhibit greater values for the number of sprints, sprint distance, and high-speed running than their counterparts in the 3-5-2 formation. This finding can be attributed to the tactical role of wide midfielders in both the 3-back and 4-back formations. Specifically, in four-back formations, the tactical role of the wide midfielder is more offensively oriented^7^. They need to sprint more often and run at high speeds to impact their opponent's defense. In contrast, in the 3-5-2 formation, wide midfielders are responsible for providing width during the attack, which means that they do not need to sprint or run at high speeds as frequently as their counterparts in four-back formations.

There are significant differences in the technical performance of wide midfielders between three-back and four-back formations. Specifically, wide midfielders in the 3-4-3 and 3-5-2 formations make more passes than their counterparts in four-back formations. Additionally, the ball retention percentage for wide midfielders is greater for the 3-4-3 formation than for the 4-4-2, 4-1-4-1, and 4-2-3-1 formations. This result can also be attributed to the tactical roles of players in different formations^5,26^, as three-back formations impose greater technical demands on wide midfielders, while four-back formations impose more physical requirements on them.

Considering the physical performance of forwards, only a few differences occur between formations. Forwards in the 4-4-2 and 3-5-2 formations cover more total distance than their counterparts in the 4-2-3-1 formation. This could be attributed to the fact that in the 4-2-3-1 formation, there is only one forward who typically acts like a center forward (known as a ‘false nine’)^26^. Specifically, this forward usually has two ways of receiving the ball. First, they can receive the ball between the two opposing central defenders, acting as the sole pivot point for their team's attack. Second, they can play in an advanced position on the pitch and have the freedom to roam and drop back to deeper positions to receive the ball, which could draw opposing center defenders out from their defensive line and thus leave space for their teammates to run in. As a result, forwards in the 4-2-3-1 formation may not need to run distances as long as those run by those in formations with two forwards (e.g., 4-4-2 and 3-5-2). This could be one of the primary reasons for our observed results.

In terms of technical performance, on the one hand, forwards in the 4-2-3-1 formation demonstrate more challenges than those in the 3-4-3, 4-3-3, and 4-4-2 formations. As mentioned above, there is only one forward in the 4-2-3-1 formation^26^. Thus, the position of this player is greater on the pitch and usually very close to the opponent's two central defenders. As a result, once there is a ball in play on the field, he or she will face intense defense from the opponent's defenders. This may be the primary explanation for why they experience more challenges. On the other hand, the number of passes made by the forwards in the 4-3-3 formation are greater than those in the 4-1-4-1 and 4-2-3-1 formations. This result may be rationally explained by the fact that the 4-3-3 formation naturally creates triangles, providing forward players with more passing options during the game and enabling them to better communicate and connect with their teammates through passing.

## Limitations

This study has certain limitations. First, when assessing the positions of players in different tactical formations, their positions were discussed only in a general sense, without a detailed distinction between offensive and defensive positions. Second, in formations with three or four defenders, labeling all wide defenders as "full-backs" may not accurately define their roles to a sufficient extent. Future research should further differentiate players based on their specific tactical roles to achieve more comprehensive and accurate analytical results. Despite these limitations, this study provides valuable insights into the impact of different tactical formations on players' physical and technical performance. Subsequent research can build upon the existing foundation and employ more refined and comprehensive methods to assess player performance.

## Conclusion

The findings of this study demonstrate that tactical formation significantly impacts the physical and technical performance of players in different positions. This outcome is primarily attributed to the different tactical roles of players within various formations. Therefore, customizing physical conditioning and technical training programs according to position and team formation is essential.

Specifically, the study reveals that the 3-5-2 and 3-4-3 formations place greater physical demands on fullbacks, while the 4-4-2, 4-3-3, 4-1-4-1, and 4-2-3-1 formations require fullbacks with greater technical ability. Additionally, wide midfielders in four-back formations exhibit superior performance in terms of sprinting and high-speed running compared to their counterparts in the 3-5-2 formation. Moreover, central midfielders in the 3-5-2 formation show a lower ball retention percentage, pass success rate, and total passes than those in the 4-4-2, 4-1-4-1, 4-3-3, and 4-2-3-1 formations. Finally, forwards experience more challenges in the 4-2-3-1 formation than in the 3-4-3, 4-3-3, and 4-4-2 formations.

In summary, this study offers valuable insights for coaches and academic staff to comprehend the physical and technical requirements of different positions across various tactical formations, thereby optimizing the training process. Future research could incorporate more technical-tactical variables to obtain a more comprehensive understanding of players' performance.

## Data Availability

The datasets generated for this work can be accessed by contacting the corresponding author upon request.
